# Assessment of aluminum bioavailability in alum sludge for agricultural utilization

**DOI:** 10.1007/s10661-017-6133-x

**Published:** 2017-07-31

**Authors:** Joanna Kluczka, Maria Zołotajkin, Jerzy Ciba, Magdalena Staroń

**Affiliations:** Department of Inorganic Chemistry, Analytical Chemistry and Electrochemistry, Faculty of Chemistry, Silesian University of Technology, B. Krzywoustego 6 Street, 44-100 Gliwice, Poland

**Keywords:** Sewage sludge, Alum sludge, Bioavailable aluminum, Soil contamination

## Abstract

Inorganic aluminum ions, [Al(H_2_O)_6_]^3+^, [Al(OH)(H_2_O)_5_]^2+^, and [Al(OH)_2_(H_2_O)_4_]^+^, are toxic to a number of crops. The aim of this study was to estimate the danger of soil contamination of bioavailable aluminum and heavy metals forms because of alum sludge which was a by-product of water, and wastewater treatment technology using aluminum coagulant is introduced into the soil. Aluminum and selected heavy metal fractionation was carried out in the post-coagulation sludge collected at a water treatment plant (where aluminum was used as a coagulant), fermented sewage sludge at a municipal wastewater treatment plant (which did not apply aluminum coagulant), and soil from water treatment plant as well as the mixtures of sludge and soil. It has been found that post-coagulation sludge used as natural fertilizer is a secondary source of bioavailable aluminum, especially when aluminum coagulants are used during water and wastewater treatment. The evaluation of applicability of the sludge to very weak acidic and acidic agricultural soils was carried out. The authors shall debate the question whether, in this case, the Regulation of EU and Polish Government on sewage sludge should also take the bioavailable aluminum into account and add to the list of the elements whose allowable contents are limited.

## Introduction

Alum sludge is the by-product generated by the water purification and wastewater treatment plants when aluminum (Al) salts are used during the coagulation process (Dassanayake et al. 2015). Wet sludge makes up to 5% in the total quantity of processed water and it is difficult and expensive to treat. The wet sludge after dewatering and drying is placed on landfills (Kyncl et al. 2012). Depending on the composition and quality of the sludge, it may be treated in different ways. In the available literature, there are numerous research studies on sewage sludge utilization (Kelessidis and Stasinakis 2012; Fytili and Zabaniotou 2008); however, review articles on alum sludge utilization are not many (Dassanayake et al. 2015; Babatunde and Zhao 2007). Traditional solutions for waste management include use as a pollutant removal agent (e.g., heavy metals adsorbents); environmental applications, for example, re-use as a coagulant in wastewater; and use in agricultural or forest sectors (Dassanayake et al. 2015). In Poland sludge, both from water purification plants and from municipal sewage treatment plants are primarily managed for agriculture and land reclamation. The importance of the problem is well illustrated in Table 1, which summarizes the figures for the last 15 years (Central Statistical Office Bochenek 2014).

**Table 1:** Sewage sludge from municipal wastewater treatment plants in Poland (Central Statistical Office Bochenek, 2014)

Specification	2000	2005	2010	2012	2013
	In thousand tonnes of dry solid per year				
Total sewage sludge generated during the year of which (%):	359.8	486.1	526.7	533.3	540.3
- Applied in agriculture	–	13.6%	20.7%	21.6%	19.5%
- Applied in land reclamation including reclamation of land for agricultural purposes	–	24.8%	10.3%	9.4%	5.4%
- Applied in cultivation of plants intended for compost production	7.1%	5.6%	5.9%	6.2%	6.0%
- Thermally transformed	1.6%	1.3%	3.8%	10.6%	13.5%
- Landfilled	42.1%	31.0%	11.2%	8.8%	5.8%
Sewage sludge accumulated on the wastewater treatment plants—as of end of the year	675.0	782.7	332.4	208.1	219.8

The regulations of the Polish Minister of the Environment of 6 February 2015 limit the amount of heavy metals (Cd, Cu, Ni, Pb, Zn, Hg, Cr) that can be discharged with municipal sewage sludge into soil annually. The pH of the soil in areas used for agriculture, on which sewage sludge is going to spread, must not be less than pH = 5.6.

Aluminum coagulants are applicable to remove colloids and phosphorus compounds in the water and wastewater treatment. Aluminum sulfate(VI), sodium tetrahydroxyaluminate, aluminum chloride, and pre-hydrolyzed aluminum coagulants are most commonly used. Although more expensive than ferric ones, aluminum coagulants are very popular especially in the drinking water treatment since they do not affect the water.

The percentage of the various forms of aluminum ions in dispensing solution coagulants 5 and 10% aluminum sulfate(VI) solution, and 5 and 10% aluminum chloride solution was estimated (Gregory and Duan 2001; Łomotowski 2007). The composition of the aqueous aluminum sulfate(VI) solution, depending on its concentration, is predominated by AlSO_4_^+^ (55–60%) and Al(SO_4_)_2_^−^ (30%) ions while the concentration of the hydrated Al^3+^ ions is approximately 10–11%. Aluminum chloride solution contains mainly the Al^3+^ form. After a coagulant is introduced into treated water or sewage, by hydrolysis of coagulant, a decrease of wastewater alkalinity is observed. New balances are established in the system and the Al^3+^ fraction depends largely on the pH of a solution. This can cause undesirable change the physico-chemical composition of purified water, and among other things, increase of the toxic aluminum ion concentration. The increased content of Al^3+^, AlOH^2+^, and Al(OH)_2_^+^ ions in the diet is a threat to human and animal health.

The resulting alum sludge can be a source of toxic aluminum and contaminate the environment. When this sludge is used for agricultural purposes, broadly defined introduces aluminum into the soil. The phytotoxic properties of aluminum with respect to primarily coniferous trees are well known. As early as 1980, Ulrich et al. (1980) and others (Smoliński et al. 2009; Yang et al. 2015) found that an increase in aluminum compounds in soil solutions was one of the main factors causing the death of forests in certain areas of North America and Europe. The toxic properties of aluminum affect not only trees (Brunner and Sperisen 2013) but also other plants grown on an industrial scale, such as tomatoes, lettuce, beetroot, alfalfa, barley, some species of grasses, and wheat (Barszczak and Bilski 1983; Samac and Tesfaye 2003). For example, the varieties that are resistant to elevated levels of aluminum, medium sensitive and those which are sensitive to concentrations of less than 2.4 mg/kg, can be distinguished between wheat varieties in aquaculture (Barszczak and Bilski 1983).

In the literature, there are papers devoted to examining the impact of the composition and structure of aluminum coagulants to residual aluminum content, i.e., introduced into the treated water with aluminum coagulant (Gregory and Duan 2001; Yang et al. 2012; Rak and Świderska-Bróż 2001; Wolborska et al. 1999). It should be noted that authors of these works had not analyzed the content of aluminum fractions, but only its total content. In Poland, this value is normalized by the Regulation of the Minister of Health of 29 March 2007 and of 20 April 2010, according to which the total concentration of aluminum ions in drinking water may not be higher than 0.200 mg/l. There are few works devoted to study whether the use of sewage sludge containing aluminum coagulant for agricultural purposes is a danger of soil contamination with toxic forms of aluminum. Codling (Codling 2008) reported that acidification of alum sludge-treated soils resulted in a larger solubility of Al in comparison to an unamended control soil. Furthermore, the phytotoxicity of aluminum is stated not only at acid but also at high pH of soil. Brautigan noticed the significant reduction of the stem and root development of field pea test plants over and above that was caused by alkalinity alone (Brautigan et al. 2012).

In terms of aluminum mobility, we can distinguish the following forms: practically unavailable and sparingly available to plants (e.g., crystalline aluminum) as well as bioavailable forms (aluminum bound with organic matter and exchangeable aluminum) (Kotowski et al. 1995). [Al(H_2_O)_6_]^3+^ aqua complex is regarded as the main phytotoxic component (Kotowski et al. 1995; Drábek et al. 2005). The literature offers numerous publications which suggest that aqua hydroxo-complexes, [Al(OH)(H_2_O)_5_]^2+^ and [Al(OH)_2_(H_2_O)_4_]^+^, have also toxic properties, and polymeric aluminum hydroxo-complexes exhibit particularly strong phytotoxic characteristics (Kotowski et al. 1995; Drábek et al. 2005; Klöppel et al. 1997; Matczak-Jon 1995; Manoharan et al. 2007; Matùš et al. 2006). On the other hand, fluoride, sulfate, and organic aluminum complexes are regarded as harmless, although there are papers which characterized the first two complexes as slightly toxic (Kotowski et al. 1995; Drábek et al. 2003; Frankowski et al. 2010).

Therefore, in order to safe alum sludge utilization in agricultural operation, such factor as aluminum toxicity should be accurately studied. The aim of this study was to estimate the danger of soil contamination of bioavailable aluminum and heavy metal forms after the alum sludge produced in wastewater treatment technology using aluminum coagulant was introduced into the soil.

For this purpose, aluminum and selected heavy metal fractionation was carried out in the post-coagulation sludge collected at a water treatment plant (where aluminum was used as a coagulant) and fermented sewage sludge at a municipal wastewater treatment plant (which did not apply aluminum coagulant). The evaluation of applicability of the sludge to very weak acidic and acidic agricultural soils was carried out.

## Experimental

Two samples of sludge were taken:

1. 40 l of post-coagulation sludge (1) was collected at a water treatment plant which used aqueous solution of aluminum hydroxide chloride (commercially named Flokor (wodkaneko.pl Product catalog 2015)) as a coagulant. The sludge was air-dried, crushed in an agalite mortar, and sieved through a 200-μm sieve.
2. 30 kg of fermented sewage sludge (2) was collected at a municipal wastewater treatment plant which employed Bardenpho technology and did not use aluminum coagulants. Similarly, the sludge was air-dried, crushed, and sieved through a 200-μm sieve.

Samples of raw water (inlet) and treated (outlet) in the water treatment plant, wherein the coagulant Flokor was used in water purification technology, were collected.

In order to find out the suitability of the sludge for fertilizing purposes, mixtures of the sludge with soil were prepared. Thirty kilograms of soil was collected from the surface layer (0–20 cm) of an allotment in Zabrze (Upper Silesia in Poland). The soil was air-dried, crushed, and sieved through a 2-mm sieve. Afterwards, the following mixtures were prepared:

1. 50 g of post-coagulation sludge (1) + 75 g of soil + 15 ml of demineralized water
2. 50 g of post-coagulation sludge (1) + 75 g of soil + 15 ml of 1 mol/l sulfuric acid
3. 25 g of sewage sludge (2) + 50 g of soil + 15 ml of demineralized water
4. 25 g of sewage sludge (2) + 50 g of soil + 15 ml of 1 mol/l sulfuric acid

The high mass ratio of the sludge to the soil resulted from the possibility for finding changes in bioavailable aluminum concentration in the mixtures in a relatively short time. After having been homogenized, the mixtures were placed in plastic containers, covered with watch glass, and stored in the dark for 9 weeks. Then, the contents of each container were dried, crushed, and sieved through a 2-mm sieve.

In addition, a soil sample was collected from the area of water treatment plant, which over the years, gradually hopping coagulant used, the purpose of its disposal.

Using known techniques (Ostrowska et al. 1991; Zołotajkin et al. 2011; Zołotajkin et al. 2014), the samples of sludges, soil from Zabrze, soil from water treatment plant as well as the mixtures of sludges, and soil described above were assayed for the concentrations of total aluminum and selected metals: zinc, lead, cadmium, nickel, chromium and copper (after a sample was digested in hydrofluoric acid), exchangeable aluminum (by sample extraction with 0.1 mol/l solution of barium chloride), and bioavailable aluminum (by sample extraction with 0.1 mol/l solution of sodium diphosphate(V)). The BCR sequential extraction procedure (Mossop and Davidson 2003) was used to assay the concentrations of zinc, cadmium, nickel, chromium, and copper in the fractions: exchangeable (by sample extraction with 0.11 mol/l solution of acetic acid), Fe and Mn oxides bound (by sample extraction with 0.1 mol/l solution of hydroxylamine hydrochloride), organically bound (by sample extraction with 30% solution of hydrogen peroxide in nitric(V) acid), and residue (after mineralization using concentrated nitric(V) acid and tetraoxochloric(VII) acid). All the analyses were repeated six times. Metal concentrations in the eluates were determined: aluminum by AAS method using Varian SpectrAA 880 and other metals by ICP OES method on Varian 710-ES. pH_KCl_ of soil samples were determined potentiometrically (Ostrowska et al. 1991).

Additional assays of monomeric and colloidal aluminum in the water samples collected at the inlet and outlet of the water treatment plant were made (Kluczka et al. 2012). The samples were filtered through a 0.45-μm filter and fixed with nitric acid up to pH = 2.0. Aluminum was determined by the spectrophotometric technique (Varian Cary 50 Scan) with eriochromocyanine R after the water samples were digested with concentrated sulfuric(VI) and nitric(V) acids (Kluczka et al. 2012). All the analyses were repeated three times.

The results are given in Tables 2, 3, 4, and 5.

**Table 2:** The content of aluminum and selected heavy metals in the post-coagulation sludge (1) and sewage sludge (2) (mg/kg)

Sludge	Aluminum fractionation		Heavy metal fractionation—BCR method						
	Fraction	Al	Fraction	Pb	Cd	Ni	Zn	Cu	Cr
Post-coagulation sludge (1)	Ext. BaCl_2_	22 ± 7	1	n.a.	0.4 ± 0.2	1.0 ± 0.4	16 ± 2	< LOD	n.a.
	Ext. Na_4_P_2_O_7_	8624 ± 458	2	n.a.	0.5 ± 0.2	0.5 ± 0.4	26 ± 7	< LOD	n.a.
			3	n.a.	2.5 ± 0.2	15 ± 0.5	32 ± 8	4.4 ± 0.3	n.a.
			4	n.a.	< LOD	17 ± 2.8	98 ± 3	34 ± 0.9	n.a.
			Sum		3.4	33	172	38	
	Mineralization	148,500 ± 155	Total content	25	4.7 ± 0.1	42 ± 1.3	157 ± 4	25 ± 0.4	27 ± 5.8
Sewage sludge (2)	Ext. BaCl_2_	6 ± 2	1	n.a.	0.95 ± 0.3	2.8 ± 1.1	418 ± 131	3.6 ± 0.5	< LOD
	Ext. Na_4_P_2_O_7_	1781 ± 176	2	n.a.	1.1 ± 0.4	1.7 ± 0.6	586 ± 182	3.2 ± 1.4	< LOD
			3	n.a.	3.0 ± 0.4	13 ± 1.5	1303 ± 214	160 ± 6.5	< LOD
			4	n.a.	< LOD	11 ± 2.2	49 ± 5	26 ± 0.8	49 ± 3.3
			Sum		5	28	2355	193	49
	Mineralization	17,150 ± 1162	Total content	60 ± 9	2.9 ± 0.8	21 ± 2.5	2050 ± 28	161 ± 7.5	48 ± 4.8
Doses allowed by EU and Poland (Minister of the Environment, 2015)		–		750	20	300	2500	1000	500

*< LOD* below the limit of detection, *n*.*a*. not analyzed

**Table 3:** The content of aluminum and selected heavy metals in the soil collected from an allotment in Zabrze (mg/kg)

Aluminum fractionation		Heavy metal fractionation—BCR method						
Fraction	Al	Fraction	Pb	Cd	Ni	Zn	Cu	Cr
Ext. BaCl_2_	17 ± 3	1	n.a.	0.4 ± 0.3	1.2 ± 0.25	57 ± 4	< LOD	< LOD
Ext. Na_4_P_2_O_7_	525 ± 10	2	n.a.	1.2 ± 0.4	0.5 ± 0.4	86 ± 8	< LOD	< LOD
		3	n.a.	0.7 ± 0.3	3.5 ± 0.4	68 ± 9	9.5 ± 1.2	< LOD
		4	n.a.	< LOD	9.1 ± 0.3	72 ± 3	18 ± 2	75 ± 6
		Sum		2.4	14	283	28	75
Total content	20,900 ± 232	Total content	80 ± 16	0.9 ± 0.2	14 ± 1.95	257 ± 10	22 ± 2	81 ± 2
The limit values in the application of sludge for agricultural purposes (Minister of the Environment 2015)			60	2	35	120	50	75
The limit values in the application of sludge for non-agricultural land reclamation (Minister of the Environment 2015)			75	4	45	220	75	150

*< LOD* below the limit of detection, *n*.*a*. not analyzed

**Table 4:** The content of aluminum in the mixture of soil and alum sludge or sewage sludge (mg/g)

Aluminum fraction	Post-coagulation sludge (1) + soil		Sewage sludge (2) + soil	
	Without acidification pH = 6.08	With acidification pH = 4.93	Without acidification pH = 5.66	With acidification pH = 4.79
Ext. BaCl_2_	10 ± 2	47 ± 8	5.2 ± 1	4.8 ± 2
Ext. Na_4_P_2_O_7_	1305 ± 196	3051 ± 308	759 ± 118	793 ± 170
Total content	62,150		15,400

**Table 5:** The content of aluminum and selected heavy metals in the soil collected from the area of water treatment plant, on which the post-coagulation sludge was spreading (mg/kg)

Aluminum fractionation		Heavy metal fractionation—BCR method						
Fraction	Al	Fraction	Pb	Cd	Ni	Zn	Cu	Cr
Ext. BaCl_2_	36 ± 6	1	n.a.	1.2 ± 0.4	1.1 ± 0.6	36 ± 6	< LOD	< LOD
Ext. Na_4_P_2_O_7_	2662 ± 126	2	n.a.	7.1 ± 0.6	34 ± 4	158 ± 7	< LOD	< LOD
		3	n.a.	2.2 ± 0.3	43 ± 6	118 ± 15	11 ± 1	< LOD
		4	n.a.	1.5 ± 0.4	26 ± 1	63 ± 3	24 ± 0.6	107 ± 6
		Sum		12.0	104	375	35	107
Total content	37,100 ± 857	Total content	55 ± 13	9 ± 0.2	97 ± 4	325 ± 6	26 ± 0.6	76 ± 7

*< LOD* below the limit of detection, *n*.*a*. not analyzed

## Results and discussion

The Flokor coagulant is a water solution of aluminum hydroxide chloride of generalized chemical formula Al_m_(OH)_3m-1_Cl·H_2_O. It finds use in the chemical treatment of industrial and municipal wastewater and technological and drinking water, as well as in the pulp and paper industry (wodkaneko.pl Product catalog 2015).

An analysis of water samples for total monomeric and colloidal aluminum showed that aluminum concentration in raw water (before treatment) was 0.13 ± 0.08 mg/l and in treated tap water, slightly increased to 0.18 ± 0.08 mg/l, but did not exceed the value of 0.2 mg/l imposed by the regulation of the Minister of Health of 20 April 2010.

Both sludges meet the requirements of the Minister of the Environment of 6 February 2015 in terms of heavy metal concentrations and can be used in agriculture (Table 2). The total concentrations of lead, cadmium, nickel, copper, and chromium were much lower than permissible ones. Another positive point is that the metals occurred primarily as residue, except for cadmium which was bound with organic matter.

Total aluminum concentration in both sludges differed markedly. There was less, almost ten times, in the sewage sludge (2) (without aluminum coagulant). The amount of exchangeable aluminum assayed was small (accuracy of the method) in both sludges. However, the concentration of bioavailable aluminum in the post-coagulation sludge (1) was considerable (8600 mg/kg).

To prepare the mixtures of soil and sludge, soil taken from the garden allotment in Zabrze was used. The pH of the soil was slightly acidic (pH = 6.30). The soil contained a lot of lead and zinc (Table 3). The residual content of heavy metals did not exceed the limit values by the regulation of the Minister of the Environment of 6 February 2015.

Nickel and copper occurred predominantly in fraction (3) bound to organic matter and (4) residue. Cadmium in fraction (2) bound with iron and manganese oxides and (3) the organically bound. Unfortunately, part of cadmium in fraction (1) exchangeable, the easiest available, was significant (17%). There was no presence of cadmium in the residue fraction. Zinc, like cadmium, is one of the most mobile elements in soil (Kabata-Pendias and Pendias 1999). The contributions of the element in the individual fractions were the following: fraction (1)—20%, fraction (2)—30%, fraction (3)—24%, and in the residue—26%. This means that as much as 74% of zinc occurred in bioavailable fractions, the presence of chromium has been found only in the residue. The aluminum content in the exchangeable and bound to organic matter fractions was low, safe for the environment.

The results of aluminum fractionation in the mixtures of the sludge with soil from Zabrze are shown in Table 4. The amount of aluminum leached with a solution of barium chloride was on the level of accuracy of the method. More aluminum leached with the solution of diphosphate(V) sodium, which was the sum of the exchangeable fraction and bound to organic matter. The latter is not phytotoxic, but is mobile and in favorable conditions (e.g., lowering of the soil pH), may pose a hazard to the environment (Kotowski et al. 1995). The results of fractionation of aluminum by acidifying a mixture of sludge with soil using sulfuric acid (VI) are also given in Table 4. In the case of a mixture of sewage sludge (2), the pH lowering from 5.66 to 4.49 did not cause a significant change in the fractions of aluminum. However, the decrease of the pH of a mixture of post-coagulation sludge (1) from 6.08 to 4.93 resulted in more than a twofold increase in the aluminum content in the fractions: exchangeable and bound to organic matter.

Since the time of exposure of the sludge to soil in the prepared mixtures was short, a soil sample was taken from the area of water treatment plant, on which over the years the post-coagulation sludge was spreading to its disposal. The soil was fractionated (Table 5). The aluminum content in the exchangeable and bound to organic matter fractions was comparable with the results conducted in the laboratory. The aluminum content in the bioavailable fraction was almost 3000 mg/kg. A high content of heavy metals, particularly cadmium, nickel, and zinc, was noted. It seems that it can be explained by soil contamination by post-coagulation sludge in which the presence of metals was stated previously (Table 2). Very disturbing is that all these three elements were present primarily in bioavailable fractions.

**Fig. 1:**
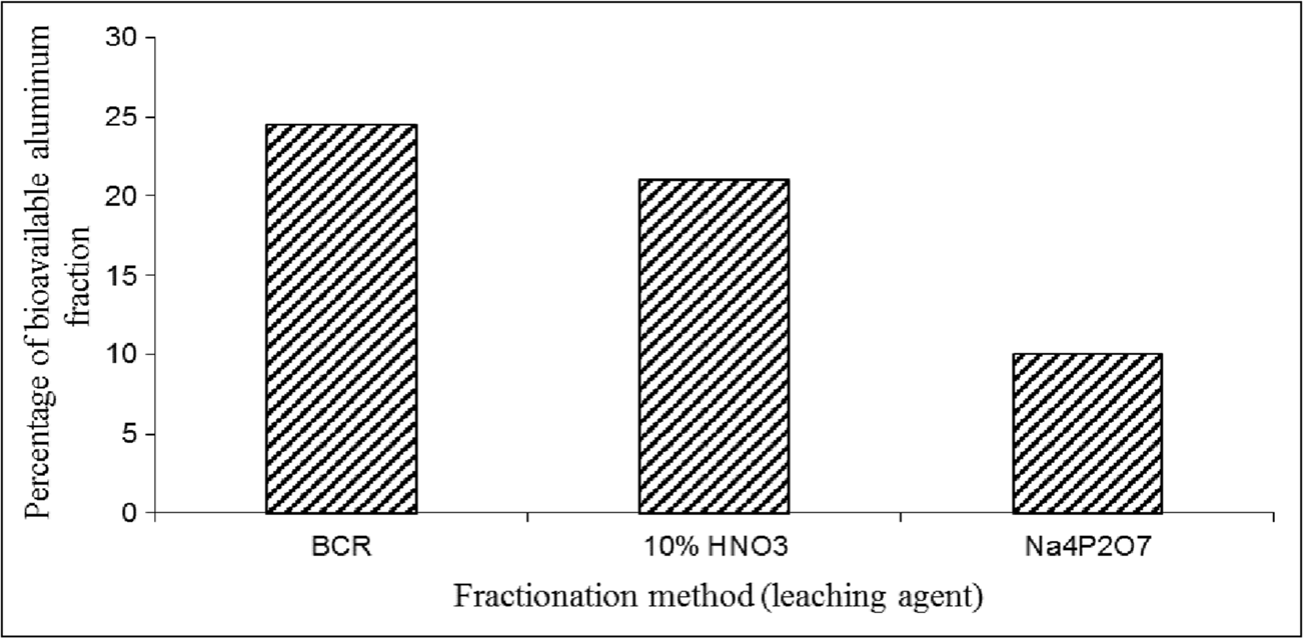
Comparison of bioavailable aluminum leached from sewage sludge (2) by BCR (a sum of fractions 1–3), 10% HNO_3_ solution, and 0.1 mol/l solution of sodium diphosphate(V)

At present, the speciation analysis of heavy metals in soil is usually made by BCR. Therefore, it seemed interesting to compare what part of aluminum can be leached using BCR (a sum of fractions 1–3) and extraction with sodium diphosphate(V) solution. Additionally, aluminum extraction with 10% HNO_3_ was compared (the method of mobile heavy metal fractions leaching from soils) (Ostrowska et al. 1991). The sample of sewage sludge (2) was used in the experiment. The results are shown in Fig. 1.

The results reveal that both BCR and 10% nitric(V) acid leach the entire bioavailable aluminum. It might enable the determination of mobile aluminum concentration without the necessity to carry out a speciation analysis of Al itself. Only when mobile aluminum concentration in the sludge was high and worrisome, the analysis should be repeated using solution of barium chloride and sodium diphosphate(V) as the extractants.

### Summary

The management of sludge has been a serious problem for years. It is used as a fertilizer in agriculture (instead of dung in short supply) due to high concentrations of organic matter and biogenic elements, such as magnesium, potassium, or calcium. However, the sludge and soil to fertilize must meet some requirements. Among other things, they must not contain large amounts of heavy metals. As demonstrated by research carried out in 1995 and then repeated in 2000 and 2005 by the Institute of Soil Science and Plant in the monitoring of arable soil chemistry, soil in Poland are not contaminated with heavy metals (IUNG-PIB Puławy). This allows a more optimistic look at the problem of the use of sludge in agriculture.

In this paper, it was stated that, according to the current metal limits, the sewage sludge in question could be used for agricultural purposes, even though it contains significant quantities of aluminum. Sequential analysis results showed that only approximately 10% of the total aluminum content of the tested sediments were as bioavailable form, which could be posed a danger to the environment. Furthermore, this is a form extracted with Na_4_P_2_O_7_ and not an exchangeable form (BaCl_2_ extractant).

The regulation of the Minister of the Environment of 6 February 2015 (according to EU) limits the content of seven metals in sewage sludge. Sobczyk (2009) notes that among the elements of which the value is limited, allowable concentrations of the Minister of the Environment of 9 September 2002 on soil and land quality standards are other elements as well: arsenic, barium, tin, cobalt, and molybdenum. Sobczyk calls for extending the list of standardized elements in sewage sludge about these five elements. In our view, it should be debated whether the aluminum should not be enclosed to this list. In this study, the post-coagulation sludge from water treatment plant was used, where newer generation coagulant is used. Unfortunately, as a coagulant, aluminum sulfate(VI) is often used. It was found that the negative effect of aluminum sulfate(VI) on the quality of treated water is greater than that of other coagulants (Wolborska et al. 1999; Nansubuga et al. 2013). It is expected that the aluminum sulfate(VI) introduced into the soil is very dangerous to the environment and will increase the concentration of toxic ions of the element.

The increase in acidity of the soil significantly increases the mobility of aluminum and heavy metals in the soil and sediments (Kluczka et al. 2012). Thus, although the regulations limit the use of sludge to soil whose pH is over 5.6, the current state of environmental pollution and the spread of acid rain to areas distant from big industrial centers suggest that bioavailable aluminum content should be also monitored.
